# Antimicrobial Action of the Cyclic Peptide Bactenecin on *Burkholderia pseudomallei* Correlates with Efficient Membrane Permeabilization

**DOI:** 10.1371/journal.pntd.0002267

**Published:** 2013-06-13

**Authors:** Kanjana Madhongsa, Supaluk Pasan, Onanong Phophetleb, Sawinee Nasompag, Sompong Thammasirirak, Sakda Daduang, Suwimol Taweechaisupapong, Andrei L. Lomize, Rina Patramanon

**Affiliations:** 1 Protein and Proteomics Research Center for Commercial and Industrial Purposes (ProCCI), Khon Kaen University, Khon Kaen, Thailand; 2 Department of Biochemistry, Faculty of Science, Khon Kaen University, Khon Kaen, Thailand; 3 Melioidosis Research Center, Khon Kaen University, Khon Kaen, Thailand; 4 Biofilm Research Group, Faculty of Dentistry, Khon Kaen University, Khon Kaen, Thailand; 5 College of Pharmacy, University of Michigan, Ann Arbor, Michigan, United States of America; Beijing Institute of Microbiology and Epidemiology, China

## Abstract

*Burkholderia pseudomallei* is a category B agent that causes Melioidosis, an acute and chronic disease with septicemia. The current treatment regimen is a heavy dose of antibiotics such as ceftazidime (CAZ); however, the risk of a relapse is possible. Peptide antibiotics are an alternative to classical antibiotics as they exhibit rapid action and are less likely to result in the development of resistance. The aim of this study was to determine the bactericidal activity against *B. pseudomallei* and examine the membrane disrupting abilities of the potent antimicrobial peptides: bactenecin, RTA3, BMAP-18 and CA-MA. All peptides exhibited >97% bactericidal activity at 20 µM, with bactenecin having slightly higher activity. Long term time-kill assays revealed a complete inhibition of cell growth at 50 µM bactenecin and CA-MA. All peptides inhibited biofilm formation comparable to CAZ, but exhibited faster kinetics (within 1 h). Bactenecin exhibited stronger binding to LPS and induced perturbation of the inner membrane of live cells. Interaction of bactenecin with model membranes resulted in changes in membrane fluidity and permeability, leading to leakage of dye across the membrane at levels two-fold greater than that of other peptides. Modeling of peptide binding on the membrane showed stable and deep insertion of bactenecin into the membrane (up to 9 Å). We propose that bactenecin is able to form dimers or large β-sheet structures in a concentration dependent manner and subsequently rapidly permeabilize the membrane, leading to cytosolic leakage and cell death in a shorter period of time compared to CAZ. Bactenecin might be considered as a potent antimicrobial agent for use against *B. pseudomallei*.

## Author Summary

*Burkholderia pseudomallei* is a category B agent that causes Melioidosis, an acute and chronic disease with septicemia. The current treatment regimen is a heavy dose of antibiotics such as ceftazidime (CAZ), however, the risk of a relapse is possible. In this study we demonstrate that bactenecin, CA-MA, RTA3 and BMAP-18 are able to inhibit the growth and biofilm formation of *B. pseudomallei*. The strong bactericidal activity of bactenecin is attributed to its greater ability to permeabilize the membrane. Computational modeling of these peptide-membrane interactions provide support for a model in which bactenecin is able to penetrate the membrane most effectively due to its cyclical structure. The peptide, bactenecin has the potential to act as a highly effective alternative to CAZ, or as a combination therapy with CAZ in the treatment of melioidosis. Furthermore, understanding the mechanism of bactenecin may help us better design more effective peptides therapeutics of choice for melioidosis.

## Introduction

*Burkholderia pseudomallei* is a category B agent and causative agent of melioidosis, a disease endemic in tropical areas such as Southeast Asia, northern Australia, the Indian subcontinent, Iran, and Central and South America, as well as other temperate regions that border the equator. *B. pseudomallei* can be found in moist soil, still water, and paddy fields and can infect humans through punctured skin or inhalation [1]. Infection of *B. pseudomallei* results in a wide range of clinical signs and symptoms, with septic shock as the most severe manifestation. Recurrence of infection is common even after treatment with a complete antibiotic regimen [2]. *B. pseudomallei* is intrinsically resistant to many antibiotics including first and second generation penicillins, cepharosporins, macrolides, rifamycins, and aminoglycosides, but is usually susceptible to CAZ, imipenem, carbapenems, amoxicillin-clavulanate, and chloramphenicol. A 20-week standard treatment includes an intravenous phase and an eradication phase through oral administration [2], [3]. The mortality rate due to melioidosis is about 20% in northern Australia and 50% overall in the less developed northeast region of Thailand [2].

Antimicrobial peptides (AMPs) are part of the innate defense system found in a wide range of organisms including insects, plants, and animals, and are used by these organisms to protect themselves from pathogenic microbe infection [4]. Unlike antibiotics, the killing mechanism of AMPs are target non-specific and usually involve membrane disruption, making them ideal for use against antibiotic-resistant pathogens [5]. AMPs generally consist of 20–40 amino acids, and are structurally organized in such a way to make them amphipathic, with one side consisting of positively charged residues and the other surface of hydrophobic residues. AMPs exhibit a broad spectrum of antimicrobial activity against Gram negative and Gram positive bacteria, fungi, and parasites. More specifically, AMPs that target Gram-negative bacteria interact with the negatively charged lipopolysaccharides (LPS) on the outer membrane via electrostatic and Van der Waals forces [6]. Examples of AMPs include human cathelicidin LL-37 [7], bovine Indolicidin [7] and bactenecin [8], bovine myeloid antimicrobial peptide-18 (BMAP-18) [9], a Cecropin A (1–8)-Magainin A (1–12) hybrid peptide (CA-MA) [10] and commensal-derived RTA3 [11].

Previous studies have reported that cathelicidin-derived peptides LL-37 and LL-31, Defensin HNP-1, histatin and histatin variants, and lactoferrin could inhibit the growth of *B. pseudomallei* [12], [13]. Comparison of the antimicrobial activity of these peptides on *B. pseudomallei* showed that the LL-31 peptides exhibited the most effective killing activity against all isolates independent of the LPS phenotype. Moreover, the LL-37 and LL-31 peptides could inhibit biofilm formation of *B. pseudomallei*. Despite the discovery of several new AMPs, little information about AMPs against *B. pseudomallei* is known. Attempts have been made in this study to search for more AMPs active against this virulent pathogen. Amongst hundreds of AMPs that have been reported to have potent antibacterial activity, four peptides were selected under the basis of having the strongest activity against *P. aeruginosa*, the most genetically related species to *B. pseudomallei*. These are bactenecin (RLCRIVVIRVCR) [8], BMAP-18 (GRWKRWRKKWKKLWKKLS) [9], CA-MA (KWKLFKKIGIGKFLHSAKKF) [10], and RTA3 (RPAFRKAAFRVMRACV) [11].

Bactenecin is a 12-aa looped antimicrobial peptide with an intracellular disulfide bond originally found in the granules of bovine neutrophils [14]. Intensive study on this short cationic peptide to modify its selectivity and activity expanded from engineering bactenecin to be in a looped or linear form to performing amino acid substitutions in order to increase or decrease the peptide hydrophobicity [15]–[17]. Recent reports also show that the oligomeric status of bactenecin determines its mode of action and dictates the strength of the antimicrobial activity [18]–[20].

Bovine myeloid antimicrobial peptide-18 (BMAP-18) is the cationic N-terminal domain of the cathelicidin member, BMAP-27, but has drastically reduced mammalian cytotoxicity [21]. BMAP-18 and its analogs exert a bacterial killing effect via forming small pores in the cell membrane, resulting in depolarization of ion flux [9]. In addition, BMAP-18 has been reported to efficiently kill African trypanosomes, fish trypanosomes and Leishmania parasites *in vitro* and also inhibit their life cycle stages [22].

Cecropin (1–8)-Magainin (1–12) or CA-MA is a hybrid peptide designed from the N-terminal cationic α-helix of cecropin A and the C-terminal amphipathic α-helix of magainin 2 [23]. CA-MA has potent antimicrobial activities against *Escherichia coli* and *Bacillus subtilis*, without hemolytic activity [24]. Analogs of CA-MA also exhibited strong antimicrobial activities due to the cationic and amphipathic structure [25], [26].

RTA3 is an amphipathic helical peptide isolated from the commensal organism, *Streptococcus mitis*. It exhibited strong broad spectrum antimicrobial activity with minimal cytotoxicity both *in vitro* and *in vivo* [27]. Thermodynamic measurements of the membrane interaction demonstrated that RTA3 and its variants show differential outer and inner membrane-specific perturbation, which explains the modulation of antimicrobial activity [11].

Here, we determine the antimicrobial activity of these peptides against *B. pseudomallei* H777, the clinical isolate from blood with high biofilm production levels [28]. We also dissected the mechanism of action of these peptides using live *B. pseudomallei*, model membranes, and molecular modeling approaches.

## Materials and Methods

### Materials

The peptides bactenecin, BMAP-18, CA-MA and RTA3 were synthesized by Tufts University Core Facility (Boston, MA) using standard fluoren-9-ylmethoxycarbonyl (Fmoc) solid-phase synthesis. The crude peptides were purified by reverse phase HPLC after which the peptides were confirmed to be greater than 99% pure by analytical HPLC and analyzed by mass spectrometry. *N*-phenyl-1-naphthylamine was purchased from Bio-Rad, USA. Polymyxin B was purchased Sigma-Aldrich, USA. Polymyxin B Bodipy FL conjugate was purchased from Molecular Probes (Leiden, The Netherlands). CAZ was purchased from GlaxoSmithKline, Italy. Luria broth and nutrient agar were purchased from Himedia, India. Formvar copper coated grids were purchased from Tedpella, USA. 96-well fluorescence plates were purchased from Corning, USA. Glutaraldehyde and formaldehyde were purchased from Poison, Australia.

### Bacterial strains and growth conditions

*Burkholderia pseudomallei* isolate H777 and *Pseudomonas aeruginosa* ATCC 27853 were used in this study. *B. pseudomallei* isolate H777 was kindly provided by Dr. Surasakdi Wongratanacheewin, Melioidosis Research Center, Khon Kaen University, Thailand. *B. pseudomallei* isolate H777 was first isolated from the blood of a patient in northeast Thailand in 2001. This wild-type isolate is characterized by the ability to produce high levels of biofilm, with a relative biofilm-forming capacity of 3.264 (OD 630) [28]. *P. aeruginosa* is a commonly found bacteria that causes disease which results in damage of tissue or inflammation and sepsis in patients with compromised immunity. *P. aeruginosa* ATCC 27853 is commonly used for antimicrobial susceptibility tests [29]. The media used in this study were Luria broth (LB) and nutrient agar (NA).The bacteria was streaked on NA and then cultured at 37°C overnight. Colonies were picked and cultured in LB at 37°C in an incubator overnight and then subcultured at 37°C in a 200 rpm shaker-incubator for 1.5 h to yield a mid-logarithmic growth phase culture.

### Antimicrobial activity

The killing activities of all peptides against *B. pseudomallei* H777 were determined by a colony culturing assay as described previously [12], [13]. Briefly, *B. pseudomallei* H777 cells were washed and resuspended to a 10^5^ CFU/mL in 1 mM potassium phosphate buffer (PPB) pH 7.0. Bacterial suspension was added at equal volumes to solutions of AMPs to attain final concentrations of 5, 10 and 20 µM for all peptides studied. Bacterial suspension without peptide was the control. Following incubation at 37°C for 1 h, the incubation mixture was serially diluted into a physiological concentration of saline and plated in triplicate on NA. Colonies were counted after 24 h of incubation at 37°C. Each assay was performed on two separate occasions, with triplicate determinations each time.

### Long-term killing kinetics

Bactericidal kinetics of the cationic antimicrobial peptides were determined using a culture of *B. pseudomallei* H777 re-suspended in 1 mM potassium phosphate buffer (PPB) at a concentration of 1×10^6^ CFU/mL [12], [13]. Each peptide was added to the bacterial suspension to a final concentration of 25, 50 and 100 µM and was incubated in a 200 rpm shaker incubator at 37°C. At indicated times (0, 1, 2, 3, 4, 5, 6 and 24 h), samples were taken, serially diluted, plated in triplicate on NA and incubated for 24 h to allow colony counting. A bactericidal effect was defined as a ≥3log_10_ reduction in CFU/mL compared to the initial inoculum.

### Prevention of biofilm formation

Effect of peptides in preventing biofilm formation was determined in 96-well microplates [30]. *B. pseudomallei* H777 were cultured in LB for 16 h and then diluted to 20% in fresh sterile medium. The cell culture was then incubated in a 200 rpm shaker-incubator at 37°C for 2 h. Cell cultures were diluted to approximately 10^6^ CFU/mL in fresh sterile medium, and each well of the microplate was filled with 100 µL of cell suspension with the peptides to a final concentration of 20, 50 or 100 µM. The plates were incubated at 37°C for the indicated times (1, 2, 3 and 4 h), after which the bacterial cells were spun and washed three times with 1 mM PPB, pH 7.0 to remove free peptide. The bacterial cells were resuspended in fresh sterile medium and cultured for another 2 days for biofilm formation. After 2 days, bacteria were washed three times with 0.9% (w/v) NaCl. The biofilm on the plates was then stained with 0.1% (w/v) crystal violet solution for 10 min, washed and then dissolved with 33% (v/v) acetic acid for 10 min. Biofilm mass was determined by measuring the absorbance at 550 nm using a microtiter plate reader.

### LPS-binding assay (PMB-BY displacement)

The binding of the cationic antimicrobial peptides to LPS was evaluated using a Polymyxin B-BODIPY (PMB-BY) displacement assay [31]. *B. pseudomallei* H777 were grown to mid-log phase in LB, spun down, and washed twice with an equal volume of 50 mM Tris-Cl, pH 7.4 and resuspended to 10^7^ CFU/mL in the same buffer. In translucent microplates, 15 µL of bacterial cells was added to 150 µL Tris buffer supplemented with PMB-BY (final concentration of 0.1 µM) and incubated for 2 h. The peptides were then (final concentrations of 5, 10, 20, and 50 µM) added and incubated further for 1 h. Samples were read on a SpectraMax M5 microplate reader (Molecular device) at an excitation wavelength of 488 nm and emission wave length of 525 nm. Maximum fluorescence was determined by measuring the fluorescence of a mixture of PMB-BY and cells without peptide, subtracting the background fluorescence of free PMB-BY in solution. Displacement of PMB-BY was measured by the reduction in maximum fluorescence due to addition of peptides. % displacement was calculated by (1-F_T_/F_0_)×100 where F_T_ is the fluorescence intensity of *B. pseudomallei* with the peptides and F_0_ is the fluorescence intensity of *B. pseudomallei* without peptides.

### Outer membrane permeability

An overnight culture of *B. pseudomallei* H777 was diluted in LB medium and grown to an OD_550_∼0.5. The cells were harvested, washed, and resuspended in the same volume of buffer (5 mM HEPES, pH 7.2, 5 mM KCN). For the NPN assay 1 mL of cells and 0.04 mM *N*-phenyl-1-naphthylamine (NPN) were mixed, and the initial fluorescence was measured using SpectraMax M5 with Ex/Em at 350 and 429 nm. Peptide was added to the mixture to a final concentration of 50 µM. Increases in fluorescence due to partitioning of NPN into the outer membrane was recorded over time until no further increase in intensity was observed. Fluorescence increase was reported as a percentage of the fluorescence measured after Triton-X 100 treatment. The fluorescence blank was NPN with bacteria only.

### Inner membrane permeability

Permeabilization of the inner membrane was investigated using an o-nitrophenyl-β-galactoside (ONPG) hydrolysis assay. Briefly [32], [33], *E. coli* MG 1655 were grown to mid-log in LB, spun down and washed twice with equal volume of PBS. Cells were diluted in PBS to 10^7^ CFU/ml (OD_550_∼0.1) was added to 135 µL of PBS supplement with 1.5 mM ONPG and 50 µM final concentration of peptides. The rate of permeability was evaluated through ONPG hydrolysis by measuring absorbance at 415 nm in SpectraMax M5 at indicated times (0, 15, 30, 45, 60 and 120 min)

### ANTS/DPX liposome leakage assay

Large unilamellar vesicles (LUVs) of egg yolk L-α-phosphatidylglycerol (EYPC): egg yolk L-α-phosphatidylcholine (EYPG) (3∶2) lipids was mixed with a dye/quencher pair 8-aminonapthalene-1,3,6 trisulfonic acid (ANTS)/p-xylene-bis-pyridinium bromide (DPX) at concentration of 12.5 mM and 45 mM, respectively, in 20 mM NaCl and 10 mM Tris/HCl, pH 7.4. The lipid suspension was freeze thawed 5× and then extruded at least 20× with a 100 nm pore polycarbonate membrane filter. After extrusion, the LUVs were isolated by gel filtration through a PD-10 column equilibrated with 100 mM NaCl and 20 mM Tris/HCl, pH 7.4 as the mobile phase. Leakage was monitored by measuring the increase in ANTS/DPX fluorescence intensity at 530 nm, with an excitation wavelength of 353 nm for 360 sec with data points taken at 20 sec intervals analyzed by SpectraMax M5. The first 100 sec of the experiments were monitored before the addition of peptide to confirm the presence of a stable baseline. A 0% dye release was determined by this initial fluorescence. The peptide was added to the sample and the fluorescent dye leakage was monitored for the remaining 360 sec [34]. The value for 100% dye release was determined by the fluorescence of the sample after addition of 0.5% Triton X-100. The percentage of leakage was calculated as: % leakage = [(F − F_0_)/(F_100_ – F_0_)]×100, where F_0_ is the fluorescence of LUV alone, F is the fluorescence intensity after peptide addition, F_100_ is the fluorescence intensity after addition of 0.5% (v/v) Triton-100 solution (complete lysis of the LUV) [35].

### Measurement of lipid fluidity

Steady state fluorescence polarization was monitored with a model Spectramax M5 to observe peptide orientation in the lipid bilayer [36]. Briefly, LUVs suspension of EYPC∶EYPG (3∶2) was mixed with 10 µM probes DPH (in dimethylformamide) to obtain a molar ratio of 10∶500 (probe/phospholipids). The DPH-anchored LUVs were incubated in the dark at room temperature for at least 1 hr and then peptides at 5, 10, 20, 25, or 50 µM was added and incubated for 30 min further. The cuvette containing the fluorescent samples was placed in a temperature controlled holder at 37°C for 10 min, and readings were taken at intervals of 2 s. The excitation and emission wavelengths were 355 and 424, respectively. The polarization values (r) of the samples were calculated by the fluorescence data manager program using the following equation:
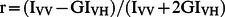
Where I_VV_ and I_VH_ are the intensities of the fluorescence (in arbitrary units) emitted parallel and perpendicular to the direction of the vertically polarized excitation light, respectively. G is the correction factor (G = I_VV_/I_HH_) for the optical system given by the ratio of the vertically to the horizontally polarized emission components when the excitation light is polarized in the horizontal direction.

### Electron microscopy

The bacterial cultures at 10^5^ CFU/mL were treated with or without 50 µM peptide for 2 h. Medium and peptide were removed by centrifuging and washing the cells in 1 mM PPB. The cells were fixed with 4% glutaraldehyde and 4% formaldehyde for 30 min, and the bacteria were dropped onto Formvar-coated grids (Tedpella, USA) and rinsed with water. The bacteria were stained with 5% uranyl acetate and silver acetate and electron micrographs were taken with a TECNAI G2 20(FEI) microscope.

### Modeling procedure

The membrane-bound α-helices of BMAP-18, RTA3, and CA-MA were generated using our program Framework/FMAP [37], [38]. The peptides were positioned in the membrane using the PPM 2.0 program [39]–[42]. The method, including calculation of protein-membrane binding affinities has been previously tested for a large set of proteins, peptides and small molecules [40]–[44]. The β-hairpin structure of bactenecin was modeled as follows. To form the disulfide bridge, two cysteine residues must be spatially close to each other on the same side of the β-hairpin. Therefore, The β-hairpin model was generated using a “non-canonical” β-hairpin template of cystatin (PDB file 1cew, fragment 50–61), which allows the arrangement of Cys^3^ and Cys^11^ residues in such a way as to form the disulfide across the non-hydrogen bonded pair of residues and the formation of a 3-residue β-turn in α_R_γ_R_β conformation. In this bactenecin model, the disulfide bridge has the gauche-gauche-gauche conformation, in agreement with experimental data [45]. After substituting all the residues in the template using QUANTA (Accelrys Software Inc.) and minimization of local energy by CHARMM (200 steps with adopted basis Newton-Raphson method, ε = 10), the models were positioned in the lipid bilayer using our program PPM 2.0 [38].

## Results

### Antimicrobial activity of peptides against *B. pseudomallei*

**Figure 1:**
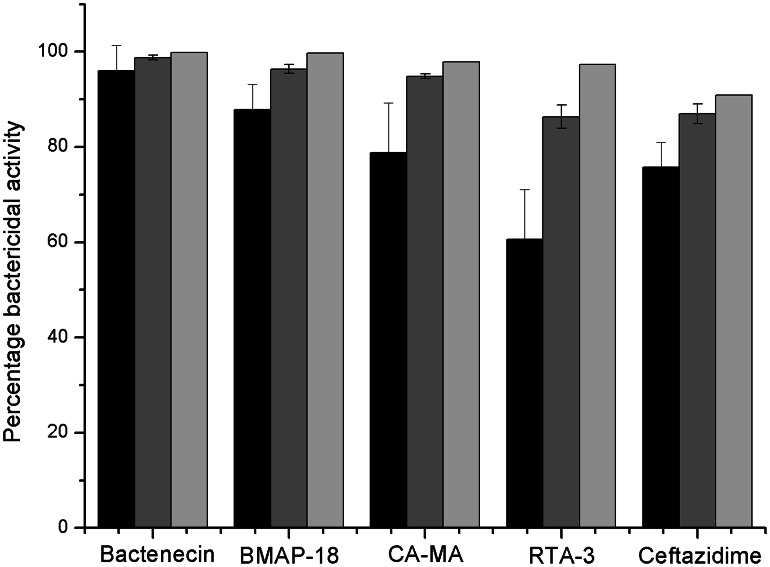
Bactericidal activities of four antimicrobial peptides against *B. pseudomallei*. Bacterial suspensions were incubated with 5(black), 10(dark grey) and 20(light grey) µM of cationic antimicrobial peptides or CAZ for 1 h. The viability of bacterial cells was determined by a plate counting technique and the data are presented as the mean and the standard deviation of two independent experiments performed in triplicate.

The bactericidal activities of cationic peptides against *B. pseudomallei* are shown in Fig. 1. At a peptide concentration of 5 µM, bactenecin exhibited the strongest (96.1%) bactericidal activity. The antibiotic ceftazidime (CAZ) resulted in only 75.4% activity, and RTA3 exhibited the lowest bactericidal activity (59.9%). When cells were treated with a higher concentration of peptide (10 µM), a greater bactericidal effect was observed for all peptides. At 20 µM, bactenecin, CA-MA, BMAP-18, RTA3, and CAZ exhibited 99.8%, 97.8%, 99.7%, 97.3%, and 90.9% killing activities, respectively.

**Figure 2:**
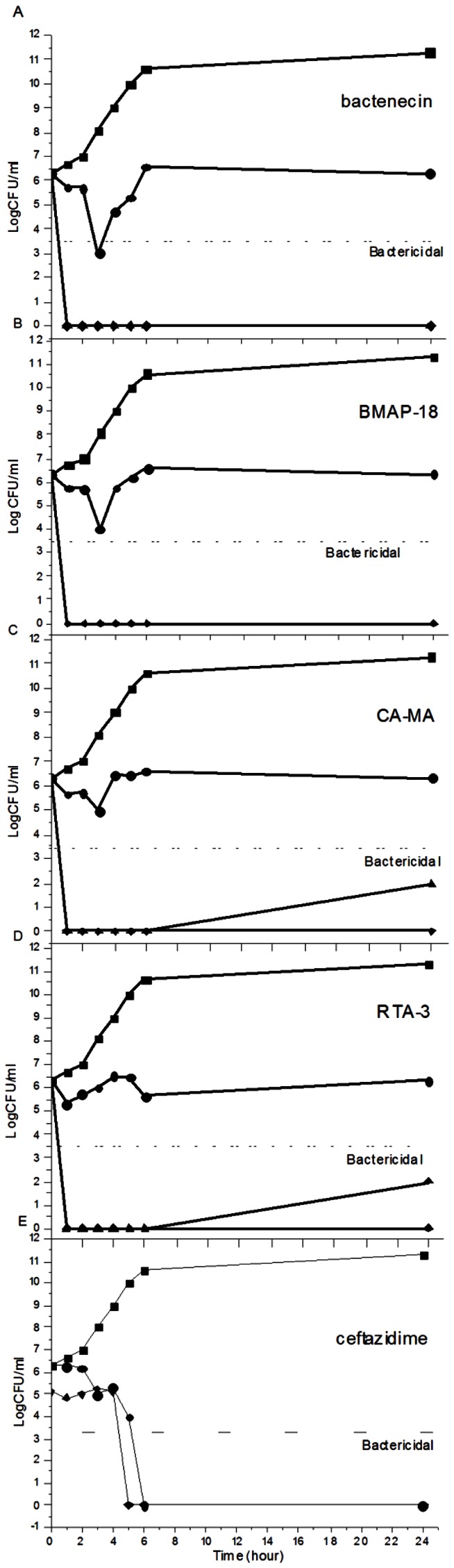
Long-term killing kinetics of four antimicrobial peptides against *B. pseudomallei*. Bacterial suspensions were incubated with bactenecin (2A), BMAP-18 (2B), CA-MA (2C), RTA3 (2D), or CAZ (2E) at concentrations of 0 (black square), 20 (black circle), 50 (black triangle), 100 (black diamond) µM and samples were taken at 1, 2, 3, 4, 5, 6, and 24 h. Colonies were counted and the bactericidal effects (dashed line) were defined as a ≥3-log reduction in colony-forming units (CFU)/ml compared to the initial inoculum. Data are the mean of two independent experiments performed in duplicate.

We next examined the long-term killing kinetics of the peptides in comparison to CAZ (Fig. 2). Under untreated conditions, the bacterial cell density increased from 6.3×10^6^ to 11.3×10^6^ CFU/mL within 24 h. Addition of 20 µM bactenecin, CA-MA, or BMAP-18 reduced the number of CFU/mL within the first 3 h, after which the bacterial cells grew to >10^5^ CFU/mL within 24 h. A complete killing was observed for cells treated with 20 µM CAZ, 50 µM bactenecin, 50 µM BMAP-18, 100 µM CA-MA, or 100 µM RTA3. Results from Fig. 1 and Fig. 2 indicate that all peptides have strong inhibition effects at early incubation times, but higher concentrations of peptide is needed to completely inhibit growth under longer incubation times. In addition, bactericidal action of the peptides appeared to be faster than that of CAZ, as seen by the complete drop in cell growth within only 1 hr at the higher concentration of peptides (Fig. 2A–D) as compared to 6 hrs for CAZ (Fig. 2E).

### Effect of peptides on prevention of biofilm formation

**Figure 3:**
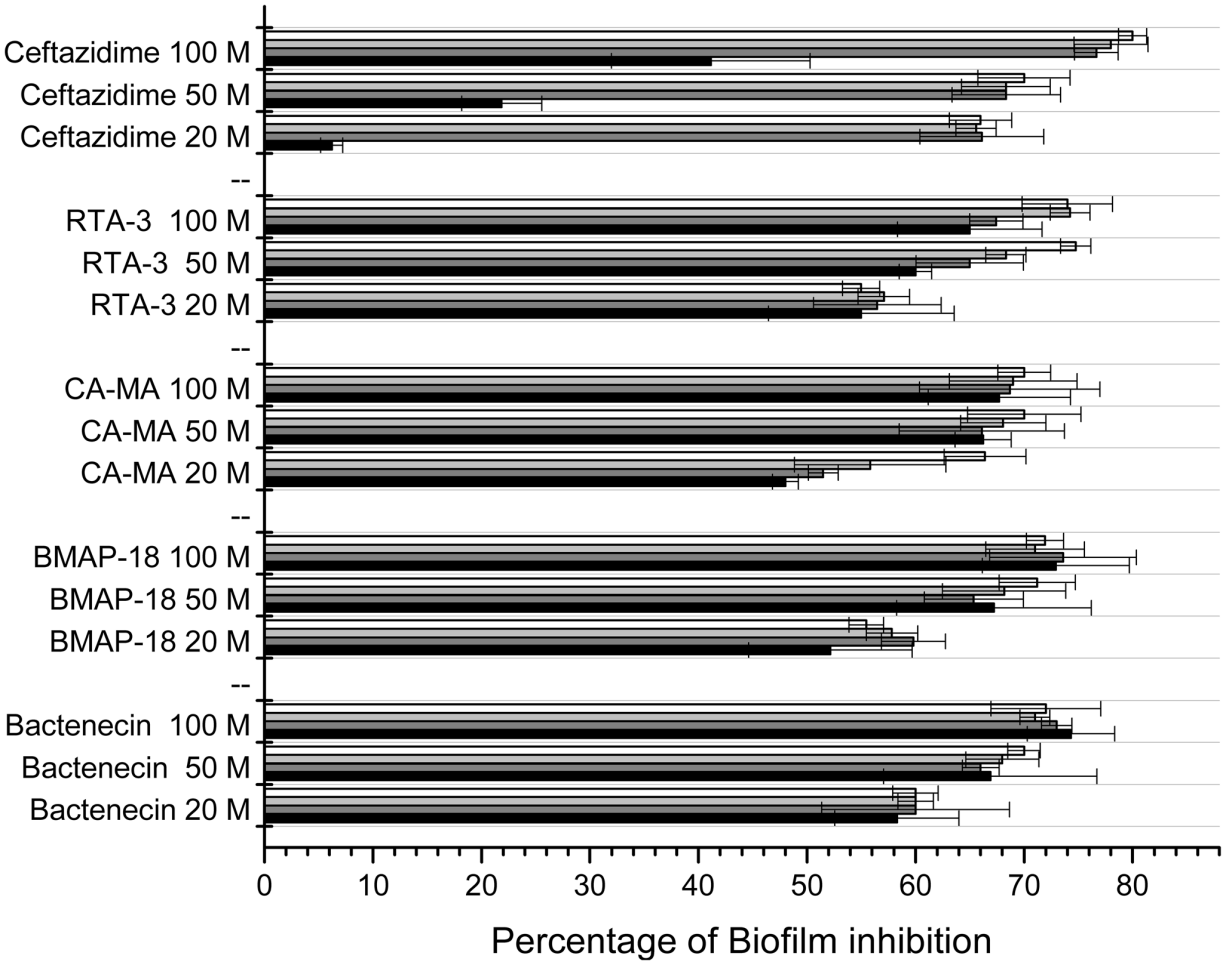
Dose and time dependent anti-biofilm activity of four antimicrobial peptides. Bacterial suspensions at 1×10^6^ CFU/ml were incubated with 20, 50 and 100 µM peptides for 1 (black), 2 (dark grey), 3 (light grey) and 4 (white) hrs at 37°C. The cells were collected and cultured for 2 days and biofilm mass was measured. The anti-biofilm activities were calculated by [(1-A_T_/A_C_)×100%], where A_T_ is the absorbance of the biofilm mass from *B. pseudomallei* treated with the peptides or CAZ, and A_C_ is the absorbance (550 nm) of biofilm mass from bacteria only. Data are the mean of two independent experiments performed in triplicate.

Biofilm formation inhibition activities of bactenecin, BMAP-18, CA-MA and RTA3 were determined and compared to that of CAZ (Fig. 3). All peptides showed more than 50% inhibition of biofilm formation at all concentrations and at all time points, except for CA-MA at 20 µM at 1 h (48.0%). Lowest inhibitory activity (48.0–58.3%) was found in cells treated with 20 µM peptide. No significant differences in biofilm inhibition were found between moderate (50 µM) and high (100 µM) concentrations of all 4 peptides (60.0–74.8% at 50 µM and 65.0–74.0% at 100 µM). The results indicate that increasing peptide concentration or increasing incubation time only slightly increased inhibitory activities. When comparing biofilm inhibition of peptides and CAZ, the inhibitory activities were not significantly different. However, CAZ appeared to have a slower inhibitory effect than that of the peptides, as we observed significantly lower activity at all concentrations after1 h incubation (6.2%, 21.8%, and 41.1% at 20, 50 and 100 µM CAZ, respectively). However, the biofilm formation inhibition activity of CAZ dramatically increased after 2 h of treatment and was stable even after longer treatment times at all concentrations tested.

### Mechanism of action of peptides against *B. pseudomallei*

**Figure 4:**
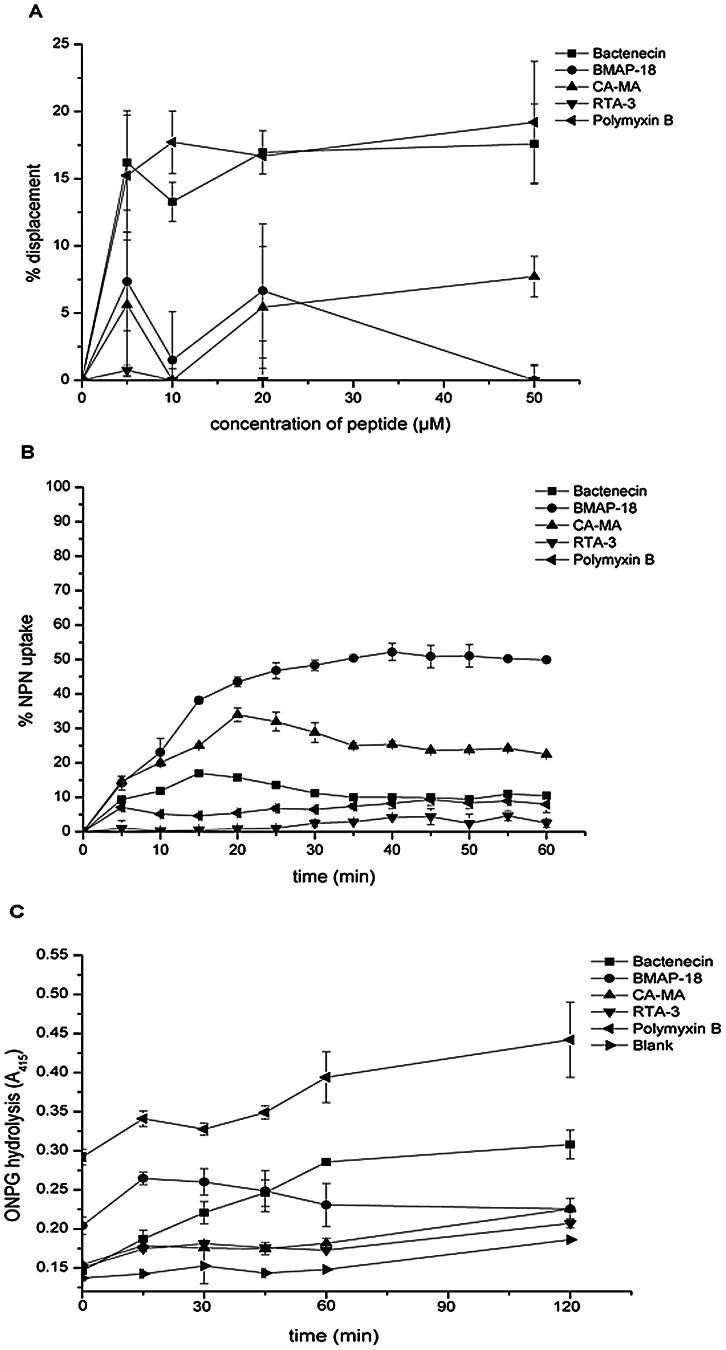
LPS binding and membrane permeabilization of four peptides on living bacterial cells. (A) LPS binding of peptides measured with a polymyxin B-BY displacement assay. *B. pseudomallei* suspensions at 1×10^6^ CFU/ml were incubated with PMB-BY for 1 hour and treated with the peptides at indicated concentrations. Fluorescence of PMB-BY was monitored 30 min after adding peptides. (B) Permeabilization of the outer membrane by the peptides was observed by NPN uptake. *B. pseudomallei* cell suspension was incubated with NPN in the presence of 50 µM peptide, and NPN fluorescence was monitored at ex/em 350/429 nm at 0–60 min. The fluorescence intensity upon peptide treatment is reported as a percentage of the maximum fluorescence intensity upon TTX-100 treatment. (C) Permeabilization of the inner membrane by the peptides was assayed by ONPG hydrolysis. The *E. coli* MG 1655 cell suspension was incubated with 50 µM peptides. Activity of leaked β-galactosidase was measured using ONPG as substrate. ONPG hydrolysis was monitored for 0–2 h. Data are the mean of two independent experiments performed in triplicate. Data are the mean of two independent experiments performed in triplicate.

In order to further determine the reasons underlying the stronger inhibitory activity of bactenecin, membrane binding and permeability of four of the peptides were compared using live cell membranes and model membranes. Initial attachment of peptide to bacterial cells involves binding of the peptide to LPS. We found that bactenecin and the control peptide antibiotic Polymyxin B (PMB) exhibited stronger LPS binding than BMAP-18 CA-MA and RTA3 (Fig. 4A). Bactenecin and PMB at 5 µM peptide concentrations resulted in 15% displacement of PMB-BY. Displacement appeared to reach saturation at 10 µM peptide, and did not increase with further addition of peptide. Similar saturation was found in BMAP-18 and CA-MA, but these two peptides caused only 5–7% displacement. RTA3 did not exhibit LPS-binding.

The ability of the peptides to permeabilize the outer membrane was observed using NPN uptake on live *B. pseudomallei*. Increase in NPN fluorescence was due to outer membrane disintegration from peptide permeabilization. The results show that the ability of the peptides to disrupt the outer membrane ranked from strongest to weakest was: BMAP-18>CA-MA>bactenecin>PMB>RTA3 (Fig. 4B). In order to complete membrane permeabilization, peptides translocation to inner membrane is one of the critical steps. We observed the ability of peptides to permeate inner (plasma) membrane using the lactose permease deficient strain *E. coli* MG 1655 [46]. Inner membrane disruption was indicated by leakage of β-galactosidase to catalyze a nonchromogenic substrate ONPG to be a yellow product ONP, as shown in Fig. 4C. Bactenecin induced inner membrane permeation at a higher rate than the three other peptides, as reflected in the greater slope of ONPG hydrolysis at 0–1 h. Permeabilization appeared to halt after 1 h. BMAP-18 showed stronger permeation than bactenecin and other peptides during the first 15 min, after which, the effect seemed to halt and slightly decrease. In sum, both assays displayed differences in the ranking of peptides in their abilities to disrupt the outer and inner membranes. The control, Polymyxin B, a well-known peptide antibiotic, exhibited differing abilities to permeabilize the inner and outer membranes as well when measured by these assays. We found that while it appeared to fail to disrupt the outer membrane, it exhibited the greatest ability to permeabilize the inner membrane in comparison to the other peptides tested (Fig. 4B–C).

**Figure 5:**
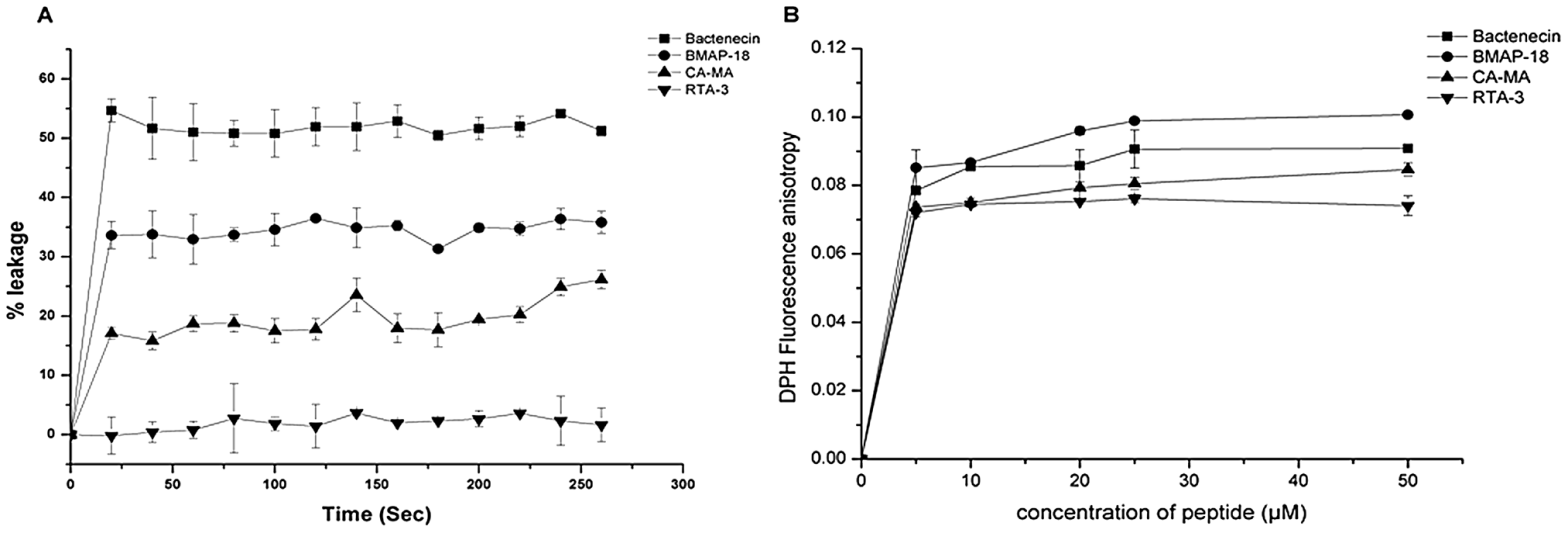
Peptide binding to model membranes resulted in membrane leakage and altered membrane fluidity. (A) Peptide binding induced leakage of ANTS/DPX-trapped model membranes was observed. After addition of 20 uM peptides into the liposome suspension, leakage was monitored by measuring an increase in ANTX fluorescence intensity at 530 nm, with the excitation at 353 nm for 360 sec. (B) hanges in fluidity of model membranes was observed. Fluorescence anisotropy of DPH anchored in LUVs (EYPG/PC 3∶1) was calculated from parallel and perpendicular fluorescence intensity values recorded at excitation 355 and emission at 424 nm, after addition of 5, 10, 20, 25 and 50 uM peptide into 10 mM HEPES buffer pH 7.4.

Next, we used model membranes to observe the direct action of the peptides on the phopholipid bilayers. LUVs were prepared containing the fluorescent probe/quencher ANTS/DPX. Leakage of the dyes was highest when the LUVs were treated with bactenecin, giving a fluorescence intensity value of about 55% the maximum fluorescence for complete dye leakage, as determined by disrupting the LUVs with TTX-100 detergent (Fig. 5A). The rest of the peptides caused dye leakage as following: BMAP-18 35%, CA-MA 22%, amd RTA3 0%. Interestingly, interaction of peptides with DPH-anchored LUVs showed fluctuation of the dye molecule in phospholipid bilayers as present in fluorescence anisotropy. The results suggest that all peptides induced changes in membrane fluidity (Fig. 5B). Taken results in Fig. 5A and Fig. 5B together, it indicated that although all peptides could perturb the membrane to an extent, bactenecin appeared to be able to induce either more or larger pores on membrane.

**Figure 6:**
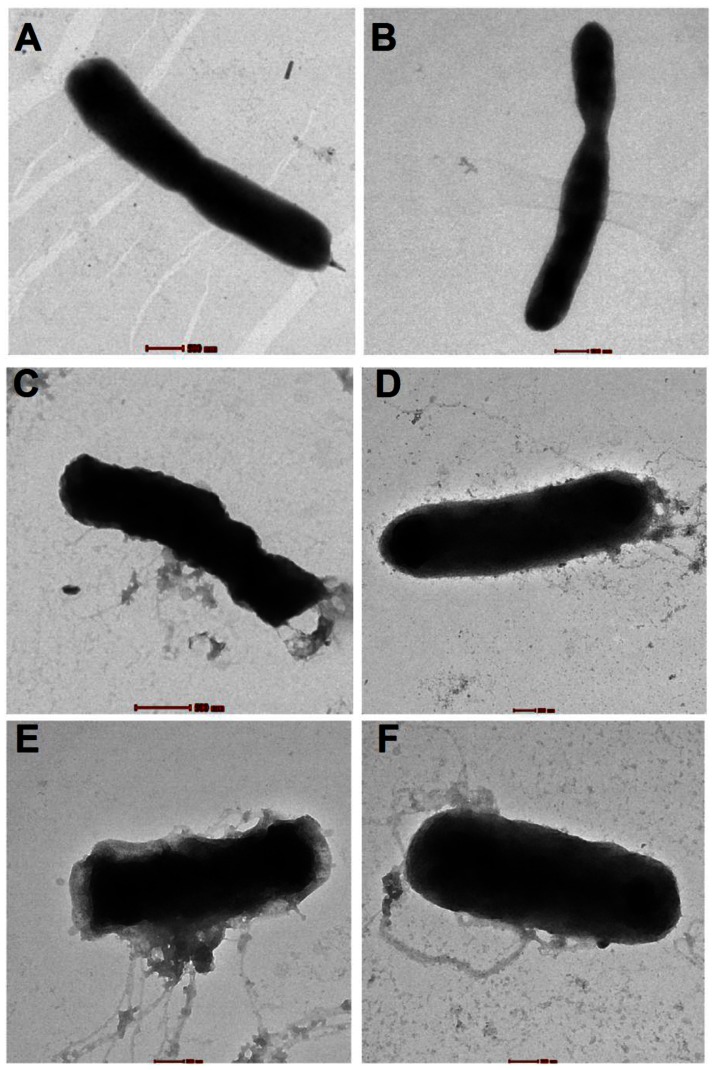
Electron micrographs of *B. pseudomallei* deformation and cytoplasmic leakage caused by four antimicrobial peptides. Bacterial suspensions at 1×10^6^ CFU/ml were incubated with or without 50 µM of the peptides for 2 h in a 37°C incubator. Untreated bacterial cells had smooth membranes and uniform shape (A) but shrunk when they were treated with CAZ (B). Cell deformation and leakage of cytosol was observed upon peptide treatment (C: bactenecin, D: BMAP-18, E: CA-MA, and F: RTA3). Scale bar represents 500 nm.

We further provide morphological evidence of these peptides acting on *B. pseudomallei* by negative staining and electron microscopy as shown in Fig. 6. Cell death caused by CAZ was characterized by cell shape deformation in the absence of cytosolic content release (Fig. 6B). In contrast, the cell death that was caused by the antimicrobial peptides was characterized by only slight cell shrinkage but with cytosolic release due to the leakiness of the membrane (Fig. 6C–F).

### Modeling of membrane-bound antimicrobial peptides

**Figure 7:**
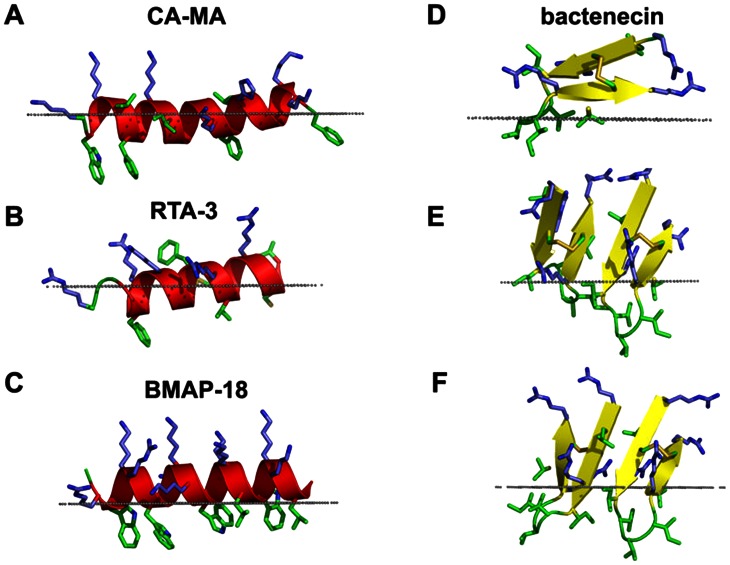
Spatial orientations of four antimicrobial peptides in membrane bilayers calculated with the PPM 2.0 program. Membrane binding modes were calculated for the predicted α-helical conformations of CA-MA (A), RTA3 (B), and BMAP-18 (C) and for the predicted β-hairpin structure of bactenecin stabilized by the disulfide Cys^3^-Cys^11^ in the monomeric form (D) and two dimeric forms: with anti-parallel pairing of the C- and N-terminal β-strands of the monomers (E) and with parallel pairing of the N-terminal β-strands of the monomers (F). Bactenecin dimer with pairing of the N-terminal β-strands showed the deepest insertion into the hydrophobic membrane core amongst the four peptides studied. Peptides are shown as ribbon diagrams colored according to secondary structure (red for α-helix, yellow for β-strand, green for loops); basic residues (Arg, Lys, His) are shown by sticks colored blue; non-polar residues (Phe, Trp, Met, Cys, Leu, Ile, Val) are shown by sticks colored green; the hydrophobic membrane boundary (at the level of the lipid carbonyls) is represented by gray dots. Images were produced using PyMol (http://www.pymol.org/).

Lastly, we performed computational modeling of peptide binding to simulated lipid bilayers (Fig. 7). Calculations using the thermodynamic model of the helix-coil transition 37,47 and our PPM program for positioning of proteins in membranes suggest that both the RTA3 and BMAP-18 peptides are unfolded in water but form an amphiphathic α-helix in the membrane-bound state. The membrane-bound cecropin-magainin hybrid peptide (CA-MA) may form either one continuous α-helix or two shorter helices separated by a Gly-Ile-Gly motif (Fig. 7A–C). Circular dichroism analysis of these peptides support the modeling results, as we found a high percentage of β-sheet in bactenecin, but high percentage of α-helix in the other three peptides in a trifluoroethanol, a membrane mimicking environment (Fig. S1 and Table S1). The calculated membrane binding energies of these peptides in the α-helical conformation were larger for BMAP-18 (ΔG_binding_ = −11.9 kcal/mol) than for CA-MA (ΔG_binding_ = −7.8 kcal/mol) and RTA3 (ΔG_binding_ = −4.2 kcal/mol). The hydrophobic region of CA-MA, including Trp3 was predicted to insert deeper into the membrane hydrocarbon core (8.2 Å from the lipid carbonyl groups) than the nonpolar residues of RTA3 and BMAP-18.

**Figure 8:**
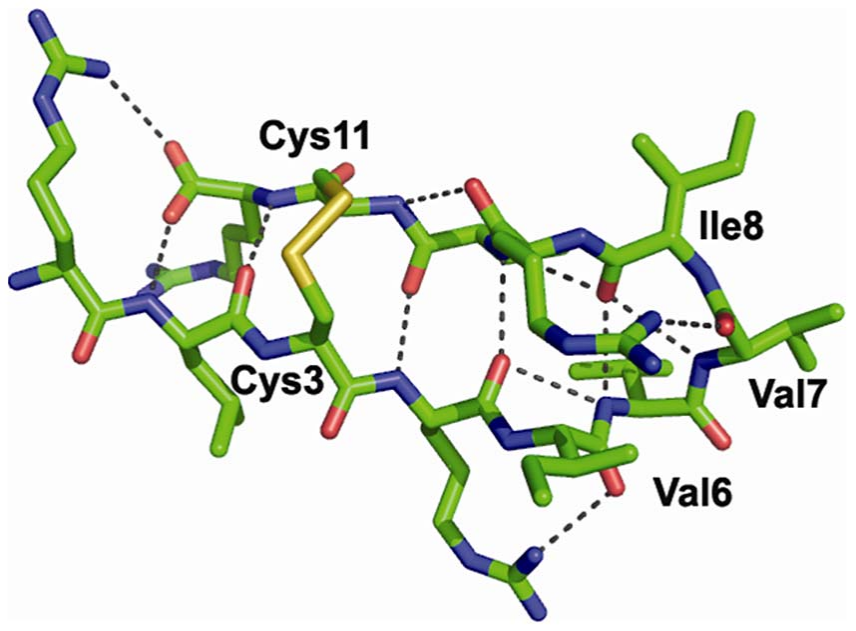
β-hairpin model of bactenecin in the oxidized state. Cys^3^-Cys^11^ disulfide bond in gauche-gauche-gauche conformation is formed in the non hydrogen-bonding pair; the 3-residue β-turn is formed by Val^6^-Val^7^-Ile^8^ residues in α_R_γ_R_α_L_ conformation [56].

Recent experimental studies indicated that Arg-rich bactenecin has a β-turn structure stabilized by a disulfide bridge in a distinct conformation that does not change significantly upon incorporation of the peptide into the lipid monolayer [45]. In addition, it has been observed that bactenecin is prone to self-association and formation of filament-like structures on membrane surfaces [45]. Therefore, we modeled the structure of bactenecin as a β-hairpin capable of self assembling into a larger β-sheet. The most probable structure of the disulfide-bridged β-hairpin with nine residues in the loop can be determined based on the requirement that a disulfide bond should be formed between the non-hydrogen-bound pair of cysteine residues in the β-sheet [48]. The β-turn portion of the loop may be composed of the Val6-Val7-Ile8 residues in an α_R_γ_R_β conformation [49] (Fig. 7D, Fig. 8). Dimerization, especially of the N/N type, allows for deeper penetration of bactenecin into the membrane core, which significantly increases peptide interactions with the membrane (Table 1). Indeed, while the bactenecin monomer was calculated to have a surface orientation on the membrane in which the maximal penetration depth of the peptide is 3.8 Å, both antiparallel and parallel dimers bind to the membrane much more tightly than the monomer (ΔG_binding_ equal to −6.6 and −8 kcal/mol for the antiparallel and parallel dimers respectively, as compared to −5.7 kcal/mol for the monomer) and are inserted much deeper into the membrane core (up to ∼9 Å depth).

**Table 1:** Parameters of spatial orientations in the lipid bilayer of antimicrobial peptides.

Peptide	Depth^a^ (Å)	Tilt angle^b^ (°)	ΔG_transfer_ (kcal/mol)	ΔG_folding_ (kcal/mol)	ΔG_binding_ c (kcal/mol)
RTA3	6.8±1.1.	82±9	−8.0	3.8	−4.2
CA-MA	8.2±3.6	85±2	−12.0	4.2	−7.8
BMAP-18	5.1±0.5	87±2	−12.3	0.4	−11.9
Bactenecin monomer	3.8±0.7	72±4	−5.7	0	−5.7
Bactenecin dimer, ↓↑	8.7±0.9	30±5	−6.6	0	−6.6
Bactenecin dimer ↑↑	8.9±0.4	59±7	−8.0	0	−8.0

The parameters were calculated by the PPM server.

a Depth of the deepest non-hydrogen atom relative to position of membrane boundary located at the level of lipid carbonyl group.

b Angle between membrane normal and main inertia axis of the molecule.

c Binding energy (ΔG_binding_) was calculated as the sum of the energy of peptide transfer from water to the lipid bilayer (ΔG_transfer_) estimated by PPM and the folding energy (ΔG_folding_) of an α-helix estimated by Framework; the folding energy of a β-hairpin stabilized by the disulfide was assumed to be zero.

## Discussion

The *Burkholderia* genus is reported to be highly resistant to antimicrobial peptides including polymyxin B [50], [51]. When comparing the antimicrobial properties of the same antimicrobial peptide towards different bacterial species, the MIC value for *Burkholderia* is several magnitudes greater than those of *Pseudomonas aeruginosa* or *Escherichia coli*. This is believed to be due to the production of a unique biofilm and the different type of LPS moiety on the *Burkholderia spp.* outer membrane. However, recent findings provide evidence of effective *B. pseudomallei* inhibition by a group of cationic peptides including LL37, Lactoferrin, and Histatin [12], [13]. Here, our study expands on these findings, and provides evidence of antimicrobial peptide efficacy against *B. pseudomallei* H777, the high biofilm-producing clinical isolate. This study demonstrates the bactericidal effects of four cationic peptides with experimental and computational evidence explaining the greater efficacy of cyclic bactenecin as compared to the linear CA-MA, RTA3 or BMAP-18 peptides. Our study appears to be the first report on antimicrobial peptides against *B. pseudomallei* that emphasizes the structure-activity relationship and provides insight into the binding mechanism of peptides on living *B. pseudomallei* and model membranes.

There are only two reports on peptides against *B. pseudomallei* that we can compare our work to [12], [13]. In our findings, low concentrations (5–20 µM) of bactenecin, CA-MA, RTA3 and BMAP-18 were able to inhibit the growth of *B. pseudomallei* close to 100%. This is a significant finding, given that in previous studies it required 100 µM of LL-37 or LL-31 for equivalent activity. However, LL-37 and LL-31 exhibited faster long-term killing kinetics than the four peptides when compared at 20 µM concentration [13]. Also, when compared with previous studies, our peptides exhibited a similar extent of biofilm inhibition as LL-37 and LL-31 [13]. With respect to mode of action, Kanthawong et al. [13] showed that LL-37 and LL-31 caused membrane blebbing when observed by freeze-fracture electron microscopy, whereas our study provides substantially more insight into the mechanism of action of the four peptides as discussed below.

The chronological events leading to bacterial killing begins with the attachment of the peptide to the outer membrane, which involves LPS-binding, followed by a cascade of events including membrane permeabilization, which leads to cell death. In PMB-BY displacement assays, bactenecin clearly showed an ability to bind LPS about 3-fold better than CA-MA, RTA3 and BMAP-18 (Fig. 4A). Once docked, all of the tested peptides caused permeability of the outer membrane to different extents, with BMAP-18 being most effective (Fig. 4B). Further disruption of the inner membrane was demonstrated to be most efficient with bactenecin at extended time points (1–2 h). BMAP-18, on the other hand, quickly penetrated the inner membrane, but the rate appeared to slightly decline at longer time points. It should be noted here that the lactose-permease-deficient strain, *E. coli* MG 1655, was used as a representative model of live *B. pseudomallei* for the inner membrane assay. Therefore, it may not directly reflect the ability of the peptides to actually cross the inner membrane of *B. pseudomallei*. We still don't have an explanation as to why bactenecin did not show effective outer membrane permeabilization but still was the most effective in the rest of the assays we tested. Therefore, in addition to observations on live *B. pseudomallei* and live *E. coli MG 1655* cells, we further investigated the peptide-membrane interaction in order to better understand why bactenecin is the most efficient peptide in killing from a mechanistic point of view. We observed the interactions between the peptides of interest and model membranes and found that bactenecin could cause almost twice as much leakage than the others (Fig. 5A). The leakage appeared not to directly correlate with greater fluidity of the phospholipid bilayers (Fig. 5B).

Taking all the evidence together, we found that stronger permeability leads to slightly better bactericidal activity (Fig. 1) of bactenecin when compared to other peptides and also CAZ. However, the difference in bactenecin effectiveness compared to the other peptides studied was most apparent in the long term time-kill assay (Fig. 2). A greater than 3-log reduction in cell growth (CFUs) was observed only after treatment of cells with 20 µM bactenecin but not the other peptides, which demonstrated strong bactericidal activity of bactenecin over extended incubation times (Fig. 2A). Moreover, a complete inhibition of cell growth was observed at 50 µM bactenecin and CA-MA, indicating that the bacterial cell killing by the peptides is concentration-dependent.

*B. pseudomallei* have been reported to form biofilm both *in vitro* and *in vivo* [52]. Previous studies have demonstrated that growing *B. pseudomallei* in biofilm stimulating conditions led to antimicrobial resistance [53] partially due to the slower diffusion of antibiotics into the biofilm layer [54]. In the present study, *B. pseudomallei* H777, an isolate categorized in the high biofilm-producing group [28], responded to the antimicrobial peptides tested faster than to CAZ. Significant biofilm inhibition was found within the first hour of treatment, demonstrating a faster killing effect of the peptides compared to CAZ (Fig. 3). This finding could be beneficial in the design of an alternative combined therapy consisting of classical antibiotics and peptide-based antibiotics.

With respect to our findings and previous studies, bactenecin appears to be the most promising peptide candidate for a synergism study. Reasons are that i) dimeric and higher ordered oligomers of bactenecin were found to be more stable than the monomeric form in both serum and high-salt concentrations, which represent physiological environments, ii) this oligomeric state of bactenecin exhibits stronger inhibitory activity [18], [19], and iii) batenecin displays faster kinetics than CAZ in long-term killing assays (our study). Therefore, we propose a melioidosis treatment regimen of combined CAZ with bactenecin, as from a mechanistic point of view, both agents would act in different manner and over a different period of time. The actions of the two therapeutics would then span a longer active period in killing *B. pseudomallei* in the circulation system of the body. Combined therapy should also result in fewer antibiotics needed and eventually lower the likelihood of possible drug resistance developed from long term therapy. However, further investigation both in the *in vitro* and *in vivo* models is required in order to explore the possibilities of the combined use of peptide and classical antibiotics in the treatment of melioidosis.

Our computational model of the β-hairpin structure of bactenecin is in good agreement with recent experimental studies of bactenecin interacting with phospholipid monolayers at the air-water interface [45]. In particular, the modeled 3-residue β-turn structure and disulfide bridges with gauche-gauche-gauche conformation conform to Raman spectroscopy studies, while the ability of these β-hairpins to form dimers and higher order oligomers as well as the predicted positions of the peptides in the membrane (Fig. 7E, 7F) are consistent with observations made in AFM studies of bactenecin-lipid films such as the formation of a filament-like network, the presence of carpet-like peptide domains, and holes in the phospholipid monolayer.

The slightly higher bactericidal activity of bactenecin (Figs. 1, 2) and its significantly greater membrane permeabilization ability (Fig. 4C, Fig. 5A) relative to other studied cationic peptides may be related to the multiple oligomerization states formed by self-associated β-hairpins of bactenecin. Each of these structural forms has a distinct mode of membrane binding and binds with variable degrees of affinity (Fig. 7, Table 1) and, therefore, differentially perturbs the lipid bilayer. The strong permeabilization ability of bactenecin is reminiscent of that of protegrin PG-1 which forms β-hairpin dimers and causes membrane thinning and torroidal pore formation [55]. However, elucidation of a detailed molecular mechanism of membrane permeabilization by bactenecin requires additional experimental and computational studies.

In conclusion, these results demonstrate that bactenecin, CA-MA, RTA3 and BMAP-18 are able to inhibit the growth and biofilm formation of *B. pseudomallei*. The strong bactericidal activity of bactenecin is attributed to its greater ability to permeabilize the membrane. Computational modeling of these peptide-membrane interactions provide support for a model in which bactenecin is able to penetrate the membrane most effectively due to its cyclical structure. The peptide bactenecin has the potential to act as a highly effective alternative to or combined with CAZ in the treatment of melioidosis. Furthermore, understanding the mechanism of bactenecin may help us better design more effective peptides that could possibly be peptide therapeutics of choice for melioidosis.

## Supporting Information

Figure S1 **CD spectra of peptides.** The secondary structure of (black square) Bactenecin, (black triangle)BMAP-18, (black circle)CA-MA, and (black diamond) RTA3 in distill water, (open square) Bactenecin, (open triangle)BMAP-18, (open circle)CA-MA, (open diamond) RTA3 in TFE.
(TIF)

Table S1 **Percentage of secondary structure of peptides.** The data was obtained from analysis of the CD spectrum with the Spectra Manager II software (Jasco J-815).
(DOC)
