# Supplementary material for: Antimicrobial Action of the Cyclic Peptide Bactenecin on Burkholderia pseudomallei Correlates with Efficient Membrane Permeabilization
Source: PLoS Negl Trop Dis. 2013 Jun 13;7(6):e2267. doi: 10.1371/journal.pntd.0002267 (PMC3681726; doi:10.1371/journal.pntd.0002267)
Supplement: Table S1 — Percentage of secondary structure of peptides. The data was obtained from analysis of the CD spectrum with the Spectra Manager II software (Jasco J-815). (DOC) [file pntd.0002267.s002.doc]

**Table S1**. **Percentage of secondary structure of peptides.** The data was obtained from analysis of the CD spectrum with the Spectra Manager II software (Jasco J-815).

| Solution | Peptides | % α-helix | % β-sheet | %β-turn | % Other |
| --- | --- | --- | --- | --- | --- |
| Distill water | Bactenecin | 5.7 | 38.0 | 11.8 | 44.5 |
|  | BMAP-18 | 27.1 | 0.0 | 21.2 | 51.7 |
|  | CA-MA | 46.3 | 0.0 | 18.7 | 35.0 |
|  | RTA3 | 41.7 | 0.0 | 19.5 | 38.8 |
| TFE | Bactenecin | 0.0 | 90.8 | 0.0 | 9.2 |
|  | CA-MA | 51.8 | 0.0 | 16.7 | 31.5 |
|  | BMAP-18 | 63.8 | 0.0 | 14.8 | 21.4 |
|  | RTA3 | 46.3 | 0.0 | 18.3 | 35.4 |
